# Possible multiple system atrophy with predominant parkinsonism in a patient with chronic schizophrenia: a case report

**DOI:** 10.1186/s12888-018-1714-y

**Published:** 2018-05-21

**Authors:** Hiroshi Komatsu, Masaaki Kato, Teiko Kinpara, Takashi Ono, Yoshihisa Kakuto

**Affiliations:** 1 Department of Psychiatry, Miyagi Psychiatric Center, Mubanchi, Tekurada, Natori, 981-1231 Japan; 2 Department of Neurology, Minami Tohoku Hospital, Iwanuma, 989-2483 Japan; 3 Department of Neurology, Kohnan Hospital, Sendai, 982-8523 Japan

**Keywords:** Multiple system atrophy, Schizophrenia, Antipsychotics, Parkinsonism, Autonomic dysfunction, Cardiac 123I-meta-iodobenzylguanidine scintigraphy, 123I-FP-CIT single-photon emission computed tomography image, Magnetic resonance imaging, Modified electroconvulsive therapy

## Abstract

**Background:**

Multiple system atrophy (MSA) is an adult-onset, rare, and progressive neurodegenerative disorder characterized by a varying combination of autonomic failure, cerebellar ataxia, and parkinsonism. MSA is categorized as MSA-P with predominant parkinsonism, and as MSA-C with predominant cerebellar features. The prevalence of MSA has been reported to be between 1.86 and 4.9 cases per 100,000 individuals. In contrast, approximately 1% of the population is affected by schizophrenia during their lifetime; therefore, MSA-P comorbidity is very rare in schizophrenic patients. However, when the exacerbation or progression of parkinsonism occurs in patients with schizophrenia treated with antipsychotics, it is necessary to consider rare neurodegenerative disorders, including MSA-P, in the differential diagnosis of parkinsonism.

**Case presentation:**

A 60-year-old female patient with chronic schizophrenia developed possible MSA-P. She had been treated mainly with typical antipsychotics, and presented with urinary incontinence, nocturnal polyuria, and dysarthria around 2011. In 2014, she developed worsening parkinsonian symptoms and autonomic dysfunction. Although her antipsychotic medication was switched to an atypical antipsychotic and the dose reduced, her parkinsonism was not improved. In 2015, modified electroconvulsive therapy produced slight improvements in the symptoms; however, she shortly returned to her symptomatic state. A combination of cardiac ^123^I-meta-iodobenzylguanidine scintigraphy and ^123^I-FP-CIT single-photon emission computed tomography imaging, in addition to brain magnetic resonance imaging findings, helped to discriminate MSA-P from other sources of parkinsonism. L-dopa had been prescribed, but she responded poorly and died in the spring of 2016.

**Conclusions:**

This case report highlights the importance of considering MSA-P in the differential diagnosis for parkinsonism in a patient being treated with antipsychotics for chronic schizophrenia. MSA-P should be considered in patients presenting with worsening and progressing parkinsonism, especially when accompanied by autonomic dysfunction or cerebellar ataxia. Although a definite diagnosis of MSA-P requires autopsy confirmation, a combination of brain magnetic resonance imaging and nuclear medicine scans may help to differentiate suspected MSA-P from the other parkinsonian syndromes. This case also demonstrates that MSA with parkinsonism that is poorly responsive to L-dopa may improve shortly after modified electroconvulsive therapy without worsening psychiatric symptoms.

## Background

Multiple system atrophy (MSA) is an adult-onset, sporadic, and rare progressive neurodegenerative disorder characterized mainly by parkinsonism that responds poorly to levodopa, cerebellar symptoms, and autonomic failure.

MSA is categorized as MSA-P when parkinsonism is predominant, and as MSA-C when associated with dominant cerebellar features. MSA-P or MSA-C is categorized by the dominant symptoms at the time of evaluation and can change by the time of diagnosis. Moreover, the diagnostic criteria for definite, probable, and possible MSA were determined at the Second Consensus Conference on the Diagnosis of MSA, held in 2007 [1].

The incidence of the disease has been estimated to be 0.6 and 0.7 cases per 100,000 persons, while the prevalence is between 1.86 and 4.9 cases per 100,000 persons [2–8]. In Asian populations, the majority of MSA cases are of the MSA-C type, whereas MSA-P type predominates in the European population [9, 10].

Schizophrenia is a chronic and serious mental disorder characterized by positive symptoms (i.e., delusions and hallucination) and/or negative symptoms (i.e., blunted affect, loss of motivation, and poverty of speech) and/or cognitive dysfunction (i.e., impairment in several cognitive domains, including working memory, attention, verbal memory and executive functions) [11]. Approximately 1% of the population is affected by schizophrenia during an individual’s lifetime, in which socio-occupational functioning is significantly impaired [12]. Antipsychotic medications are effective for relieving both positive and negative symptoms, but can cause extrapyramidal symptoms (EPS) (i.e., parkinsonian symptoms, dystonia, akathisia, and tardive dyskinesia) as a side effect. It is important to note that EPS tend to be more associated with typical antipsychotics than atypical antipsychotics [13].

Herein, we report a case involving a 60-year-old female patient with schizophrenia being treated with long-term antipsychotic medication, who developed possible MSA-P. We adhered to the CARE guidelines/methodology, which was created by an international group of experts that helps authors reduce bias and increase the accuracy, transparency, and usefulness of case reports (http://www.care-statement.org/).

## Case presentation

The patient was the first-born of 4 children, with no family history of psychiatric illness and no developmental delay. In 1972, she first presented hallucination-delusions at 16 years of age and was diagnosed with schizophrenia in another hospital. After being hospitalized two times there, she visited the Miyagi Prefecture Center (MPC, Japan) at 25 years of age in 1981. An acute change in her emotions, behavior, and thoughts occurred a few days before the first consultation at the MPC.

**Fig. 1:**
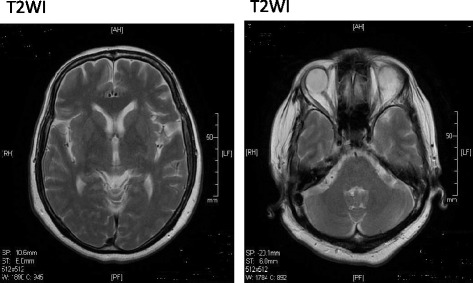
Brain magnetic resonance images of the patient at 57 years of age (2013). T2-weighted images (T2WI) demonstrated no remarkable abnormalities, including fresh cerebral infarction, bleeding, space-occupying lesions, and prominent atrophic changes in brain regions including the bilateral putamen. Moreover, there was no high-intensity signal of the lateral rim of the putamen

**Fig. 2:**
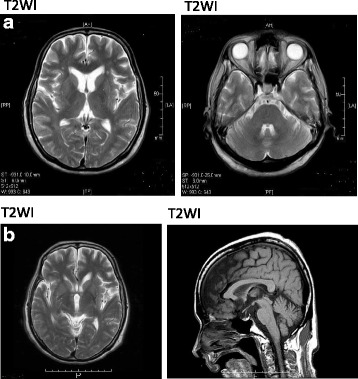
Brain magnetic resonance images of the patient at 58 years of age (spring 2015) (**a**) and 59 years of age (winter 2015) (**b**). **a** Putaminal atrophic change and low- and high-intensity signals of the lateral rim of the putamen were observed predominantly in the left putamen in the T2-weighted image (T2WI). However, T2WI did not show the hot-cross bun sign, which may reflect pontine atrophy. **b** As in the previous year, magnetic resonance imaging findings revealed putaminal atrophic change and both low- and high-intensity signals of the lateral rim of the bilateral putamen, again predominantly in the left putamen. There were slight atrophic changes in the cerebellum and brain stem

**Fig. 3:**
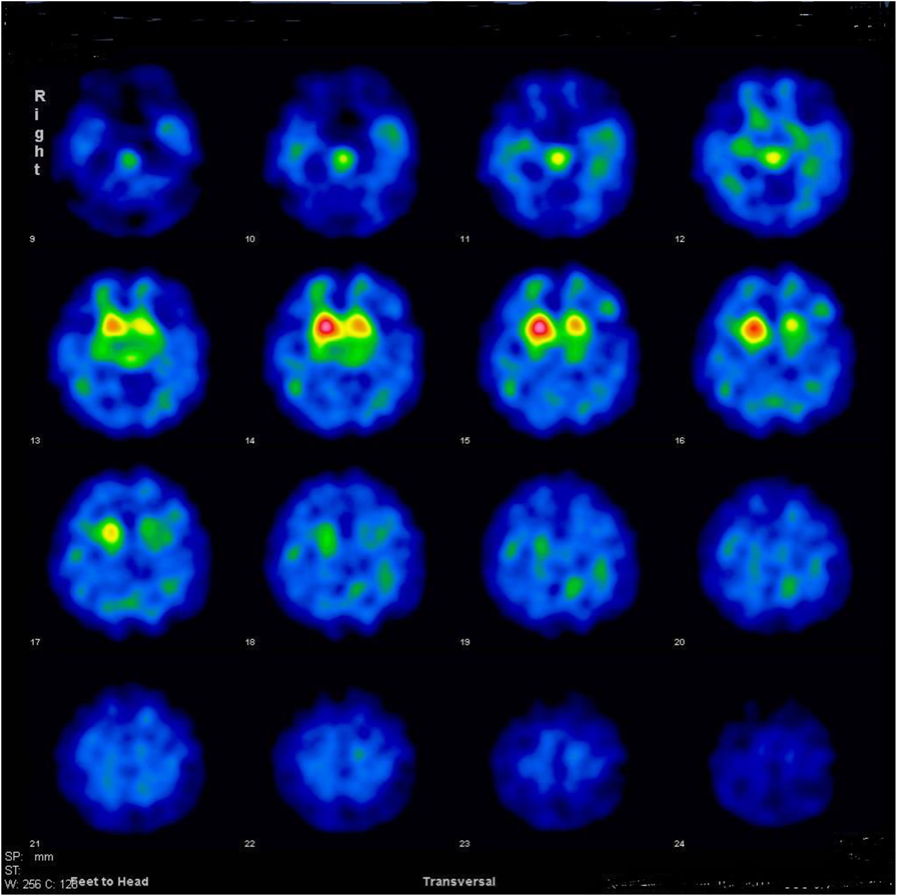
^123^I-FP-CIT (DaTscan) SPECT image results in 2015. DaTscan data demonstrate reduced bilateral striatal ioflupane (^123^I) uptake predominantly in the dorsolateral striatum. There were asymmetrical changes, with a greater decrease in ^123^I uptake in the left dorsolateral striatum

**Fig. 4:**
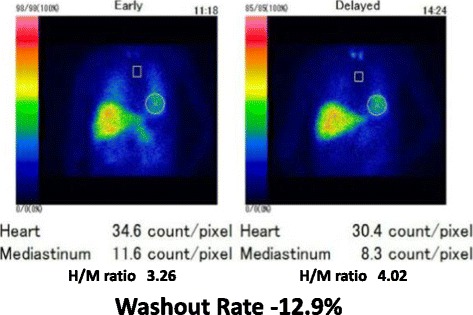
Cardiac ^123^I-meta-iodobenzylguanidine (MIBG) scintigraphy results in 2015. The values of the heart to mediastinum (H/M) ratio in the early and delayed phases were 3.26 and 4.02 (normal range is > 2.20), respectively. The washout rate was − 12.9% (normal range is < 22%). ^123^I-MIBG scintigraphy showed normal cardiac MIBG uptake

At the first examination, she was very talkative and presented severely disorganized thoughts and behavior, as well as inappropriately expressed emotions. She was readmitted to the hospital for treatment of psychotic episodes. After discharge, she was continuously treated as an outpatient; however, symptoms including avolition, asociality, moderately disorganized thinking, and emotional vulnerability to psychological stress in daily life persisted. She married at 28 years of age in 1984, and gave birth to a boy the following year. She was maintained in a generally stable mental state with the support of a community health nurse and her family. Long-acting injectable (LAI) fluphenazine enanthate was introduced in 1993 to overcome poor adherence when she was 37 years of age. At approximately the same time, she was treated for diabetes. In 1994, at 38 years of age, an overnight stay with her sister-in-law resulted in rehospitalization for a worsening of disorganized thought, anxiety, and impatience. After being admitted for a few weeks, she regularly visited our hospital as an outpatient. In 2004, she transiently presented with dizziness from standing up too quickly at around 48 years of age. In 2011, she began complaining of urinary incontinence, nocturnal polyuria, and dysarthria at approximately 55 years of age; however, it was unlikely that such symptoms were side effects of her antipsychotics or diabetic autonomic neuropathy. When she was 56 years of age, she gradually developed pain in the right knee and was diagnosed with osteoarthritis of the right knee. In the following year, she underwent artificial joint replacement surgery for her right knee in the Department of Orthopaedic Surgery, Tohoku Rosai Hospital. Bilateral hand muscle weakness, predominantly in the right hand, and dysarthria were observed while she was hospitalized in 2013. Although she underwent brain magnetic resonance imaging (MRI), findings revealed no remarkable abnormalities (Fig. 1). She used a pick-up walker at home after the knee surgery; however, at the end of the same year, she fell at home and was diagnosed with a right femoral neck fracture. She was treated operatively for the fracture in the same orthopedic surgery department as before. After 4 months as an inpatient, she was discharged to her home, and resumed outpatient treatment at the MPC in 2014. She presented with depressed mood, decreased appetite, and body weight loss after discharge, and was prescribed sertraline for symptoms of depression. In 2014, at 58 years of age, she underwent video fluoroscopic examination of her swallowing, demonstrating increased dysphagia, bradykinesia, and upper-limb muscle rigidity, predominantly in the right limb. Drug-induced parkinsonism was suspected based on the results of the examination, which indicated no organic abnormality. LAI fluphenazine enanthate was discontinued, and she was switched from oral haloperidol to aripiprazole. She was readmitted to our hospital at 58 years of age upon appearance of emotional instability, hallucinations, and negativism following the drug switch. Moreover, she also presented with worsening of urinary incontinence and retention, which required urethral catheterization. The volume of post-catheterization urinary output was greater than 600 mL. Dosage adjustment of the antipsychotics, including tests with aripiprazole, zotepine, blonanserin, and perospirone, failed to improve the parkinsonism, mutism, and negativism (e.g., food and drug rejection); consequently, modified electroconvulsive therapy (mECT) was administered for 20 treatments. mECT produced slight improvements in the symptoms, and it became possible for her to ingest a small amount of food and speak a little (2015). However, she returned to her symptomatic state after a short period of time. She then underwent careful examination for persistent parkinsonism and autonomic dysfunction (including urinary retention, urinary incontinence, and constipation) in the Department of Neurology, Kohnan Hospital (2015). Brain MRI revealed a T2-hyperintense lateral rim and hypointense putamen that had progressed over the year (Fig. 2). Cardiac ^123^I-meta-iodobenzylguanidine (MIBG) scintigraphy demonstrated normal myocardial MIBG uptake (Fig. 3). In ^123^I-FP-CIT (DaTscan) single-photon emission computed tomography (SPECT) imaging, decreased uptake of ^123^I-FP-CIT was observed in the bilateral striatum, but predominantly in the left dorsolateral striatum (Fig. 4). MSA-P was thus suspected from the clinical features (including parkinsonism, urinary dysfunction, and constipation), neuroimaging, and nuclear medicine findings. L-dopa was prescribed and the dose was increased to 700 mg/day; however, her response was poor, and she died of aspiration pneumonia in 2016 at 60 years of age.

## Discussion

MSA is a rare neurodegenerative disorder in general, and MSA-P is significantly less common than MSA-C in the Japanese population [9, 10]. In fact, to our knowledge, this is the first report to describe a 60-year-old female patient with chronic schizophrenia with possible MSA-P.

In 1900, Joseph Jules Dejerine and André Thomas first reported two cases presenting cerebellar ataxia and pathological lesions in the cerebellum and brainstem, which they described as olivopontocerebellar atrophy [14]. Subsequently, Shy and Drager described two patients with parkinsonism and autonomic symptoms including orthostatic hypotension in 1960 [15]. Van der Eecken et al. reported cases with parkinsonism and striatonigral degeneration at autopsy in the same year [16]. In 1969, Graham and Oppenheimer first introduced the term ‘MSA’ to represent all three diagnoses.

Papp et al. demonstrated the presence of argyrophilic glial cytoplasmic inclusions in the central nervous system of patients diagnosed with MSA in 1989 [17]; these inclusions were later reported to be positive for α-synuclein in 1998, and recent studies provide evidence for classifying MSA as an α-synucleinopathy, together with Parkinson’s disease and dementia with Lewy bodies [18, 19].

The first consensus conference reported by Gilman et al. categorized MSA into MSA-P and MSA-C [20]. Moreover, the conference defined diagnostic criteria to define three levels of certainty: possible, probable, and definite. The second consensus conference, held in 2007, revised the diagnostic criteria such that the criteria for probable MSA were simplified, requiring at least one feature suggesting autonomic dysfunction; also, additional features for the diagnosis of possible MSA, and features supporting MSA (red flags) were introduced (Table 1) [1].

**Table 1:** Diagnostic Criteria for definite, probable, and possible multiple system atrophy (MSA) [1]

Definite MSA	
• Neuropathological demonstration of α-synclein-positive glial cytoplasmic inclusions with neurodegenerative changes in striatonigral or olivopontocerebellar structures	
• A sporadic and progressive adult onset (>30 y) disease characterized by the symptoms below	
Probable MSA	
• Autonomic dysfunction including urinary dysfunction (inability to control the release of urine from the bladder, with erectile dysfunction in males) or orthostatic hypotension (decrease of blood pressure within 3 min by at least 30 mm Hg systolic or 15 mm Hg diastolic	
• Plus parkinsonism (bradykinesia with rigidity, tremor, or postural instability), which responds poorly to L-dopa, or cerebellar symptoms (gait ataxia with dysathria, limb ataxia, or cerebellar oculomotor dysfunction)	
Possible MSA	
• Parkinsonism (bradykinesia with rigidity, tremor, or postural instability) or same cerebellar symptoms as Probable MSA	
• Plus at least one feature suggesting autonomic dysfunction (otherwise unexplained urinary urgency, frequency or incomplete bladder emptying, erectile dysfunction in males, or orthostatic hypotension not meeting criteria of Probable MSA) and at least one of the additional features below	
Additional features of Possible MSA	
MSA-P or MSA-C	
• Babinski sign with hyperreflexia	
• Stridor	
Possible MSA-P	
• Rapidly progressive parkinsonism	
• Poor response to L-dopa	
• Postural instability within 3 y of motor onset	
• Gait ataxia, cerebellar dysarthria, or cerebellar oculomotor dysfunction	
• Dysphasia within 5 y of motor onset	
• Atrophy in MRI of putamen, middle cerebellar penducle, pons, or cerebellum	
• Hypometabolism in putamen, brainstem, or cerebellum on FDG-PET	
Possible MSA-C	
• Parkinsonism (bradykinesia and rigidity)	
• Atrophy in MRI of putamen, middle cerebellar penducle, or pons	
• Hypometabolism in putamen on FDG-PET	
• Presynaptic nigrostriatal dopaminergic denervation in SPECT or PET imaging	
Features supporting (red flag) and not supporting a diagnosis of MSA	
Supporting features	
• Orofacial dystonia	
• Disproportionate antecollis	
• Severe dysphoria	
• Contractures of hands or feet	
• Inspiration signs	
• Camptocormia (severe anterior flexion of the spine) and/or Pisa syndrome (severe lateral flexion of the spine)	
• Severe dysarthria	
• New or increased snoring	
• Cold hands and feet	
• Pathologic laughter or crying	
• Jerky, myoclonic postural/action tremor	
Nonsupporting features	
• Classic pill-rolling rest tremor	
• Clinically significant neuropathy	
• Hallucination not induced by drugs	
• Onset after age 75 y	
• Family history of ataxia or parkinsonism	
• Dementia (for DSM-IV)	
• White matter lesions supporting multiple sclerosis	

*MSA* Multiple system atrophy

*MSA-P* Multiple system atrophy with predominant parkinsonism

*MSA-C* Multiple system atrophy with predominant cerebellar features

*MRI* Magnetic resonance imaging

*FDG* [18F]fluorodeoxyglucose

*PET* Positron emission tomography

*SPECT* Single photon emission computed tomography

*DSM-IV* Diagnostic and Statistical Manual of Mental Disorders, Fourth Edition

Patients with MSA-P exhibit early development of autonomic dysfunction and more rapid progression than those with Parkinson’s disease. Low et al. reported that the mean remaining lifespan ranged from 6.2 to 10 years after symptoms first appear [21–25]. The most common causes of death have been reported to be nocturnal sudden death and aspiration pneumonia [26].

Autonomic features are a key characteristics in patients with MSA [8]. The urogenital and cardiovascular systems are most frequently affected. Criteria for diagnosis of probable MSA according to Gilman et al. (Table 1) include autonomic dysfunction including urinary incontinence (inability to the release of urine from the bladder or orthostatic hypotension (decrease of blood pressure within 3 min of standing by at least 30 mmHg systolic or 15 mmHg diastolic). Orthostatic hypotension has been reported to be apparent in 43 to 81% of patients with MSA [24, 27–30].

Olfactory function has been reported to be preserved or mildly impaired in MSA, in contrast to a marked impairment in Parkinson’s disease [31–37]. Olfactory function is evaluated with odor discrimination, threshold detection, or identification. Combination of assessment of olfactory function and other biomarkers including Cardiac MIBG Scintigraphy can be useful to discriminate Parkinson’s disease from atypical parkinsonian disorders [33, 38, 39].

Cerebellar ataxia in MSA demonstrates a poor response to medical treatment; however, in approximately 30 to 40% of patients with MSA, parkinsonism may be at least transiently responsive to L-dopa therapy in the early stages of the disease [40, 41]. However, patients with MSA are typically less responsive to L-dopa therapy than those with Parkinson’s disease.

Brain MRI may be a useful tool to distinguish MSA from other parkinsonian syndromes. Brain MRI in patients with MSA frequently reveal atrophic changes in the cerebellum, pons, and putamen. While middle cerebellar peduncle and cerebellar atrophy is more common in MSA-C than in MSA-P, putaminal atrophy is more pronounced in patients with MSA-P, which is consistent with the clinical symptoms [42]. Low intensity in the dorsolateral putamen and a slit-like, highly intense signal in the lateral margin of the putamen are commonly observed in T2-weighted MRI (T2WI) of MSA-P [42, 43]. In contrast, in addition to atrophic changes in the inferior or middle cerebellar peduncle, hot-cross bun sign, which may reflect pontine atrophy, is more commonly found in T2WI of MSA-C [8].

Nuclear medicine scans have been reported to be useful in the differential diagnosis of parkinsonism. In contrast to patients with Parkinson’s disease and dementia with Lewy bodies, patients with MSA often exhibit normal or only mildly reduced cardiac MIBG uptake in both the early and delayed image phases in MIBG-uptake cardiac scintigraphy [44, 45]. Reduced striatal ^123^I-FP-CIT uptake is observed in ^123^I-FP-CIT (DaTscan) SPECT imaging of Parkinson’s disease and other atypical parkinsonian diseases such as MSA [46]. Moreover, there are also a relatively limited number of studies reporting reduced DaTscan caused by excessive post-synaptic dopamine D2 blockade due to antipsychotic medication activity or even by the disease itself [47–53].

Clinical features and brain MRI findings together may help to differentiate MSA-P from progressive supranuclear palsy (PSP) and corticobasal degeneration (CBD). Patients with PSP develop vertical supranuclear eye movement disturbances, and exhibit atrophy of the mesencephalic tegmentum, superior colliculus, and superior cerebellar peduncle on brain MRI [54, 55]. However, patients with CBD typically exhibit pronounced frontoparietal asymmetrical atrophy that is particularly distinctive near the central sulcus in brain MRI [55].

To our knowledge, there have been no consistent reports to date demonstrating the effect of antipsychotic treatment on putaminal structure. Recent large-scale, multisite, subcortical brain volumetric studies revealed larger putaminal volume in schizophrenia compared with healthy subjects [56, 57]. Therefore, brain MRI findings, including putaminal atrophy, do not appear to be attributed to antipsychotic medications and schizophrenia itself.

Our patient developed autonomic dysfunction, including urinary incontinence and nocturnal polyuria, at around 55 years of age (2011). By 58 years of age (2014), she exhibited progression of her autonomic dysfunction (i.e., otherwise unexplained urinary incontinence and retention) and parkinsonian symptoms (i.e., postural instability, bradykinesia, and limb muscle rigidity). Consistent with previous reports, she exhibited no clinical symptoms suggesting olfactory dysfunction, although no objective test could be performed (e.g., odor discrimination test, odor identification test, etc.) because she was not able to follow instructions in the inspection test given her worsening psychiatric symptoms. Her parkinsonism did not improve after altering her dose and type of antipsychotics, and brain MRI findings revealed a T2-hyperintense lateral rim and hypointense putamen (Fig. 2). Although her cardiac MIBG scintigraphy results were normal, she exhibited decreased uptake of ^123^I-FP-CIT in the bilateral striatum, although predominantly in the left dorsolateral striatum. Finally, her parkinsonian symptoms responded poorly to L-dopa therapy (700 mg/day). This combination of clinical features, and neuroimaging and nuclear medicine results, led us to diagnose possible MSA-P.

Notably, her parkinsonism symptoms slightly improved after a number of mECT treatments; this is consistent with previous reports describing the efficacy of mECT against parkinsonism [58, 59].

Psychiatric symptoms, including emotional incontinence, depression, and anxiety, can appear in patients with MSA: 39 to 62% of patients with MSA have been reported to experience mild to severe depression. A few case reports of MSA patients developing psychosis have also been published [60–67]. Papapetropoulos et al. reported that 9.5% of patients with MSA-P experienced hallucinations [65]. There is recent, increasing evidence from brain imaging, genetic association studies, and postmortem brain studies that oligodendrocyte dysfunction is implicated in the pathogenesis of both schizophrenia and major depressive disorder [68–70]. In patients with MSA, variable degrees of olivopontocerebellar atrophy and striatonigral degeneration are typically found at postmortem confirmation, which broadly reflect the cerebellar ataxia and parkinsonism [8]. In 1989, Papp et al. demonstrated the presence of argyrophilic oligodendroglial cytoplasmic inclusions in the central nervous system of patients diagnosed with MSA [17]; these inclusions were later reported to be positive for α-synuclein in 1998. The density of α-synuclein-positive oligodendroglial cytoplasmic inclusion broadly reflects the distribution of neurogenerative changes in patients with MSA [8]. Moreover, dopamine and norepinephrine were found to be profoundly depleted in various brain regions of patients with MSA, especially in the corpus striatum, nucleus accumbens, substantia nigra, locus coeruleus, hypothalamus, and septal nuclei [71]. These findings indicate that depression and psychosis in patients with MSA may be attributed to oligodendrocyte dysfunction and catecholaminergic disturbance(s).

Our patient presented with depressive symptoms and worsening of psychosis with progress of MSA. Although it was difficult to distinguish worsening of schizophrenia from depression and psychosis development by progression of MSA, these affective and psychotic symptoms may be caused partly by oligodendrocyte dysfunction and catecholaminergic disturbance(s) accompanied by progression of MSA. The exact neuropathology of schizophrenia and depression remain unclear. Therefore, it may be worthwhile to investigate the biological mechanisms underlying psychosis and depression in patients with MSA to elucidate a patho-etiology for these diseases.

Finally, there are some limitations to our study that should be addressed. First, definite MSA requires postmortem (autopsy) confirmation of α-synuclein-positive inclusions in oligodendrocytes. However, we could not confirm a diagnosis of definite MSA due to the lack of qualified pathologists/medical doctors able to perform autopsy in our hospital. Second, we could not confirm whether significant orthostatic hypotension met the diagnostic criteria for MSA in the head-up tilted position, despite the patient’s complaints of transient dizziness from standing up. Third, we could not perform any objective urological evaluation (e.g., urodynamic testing, or post void residual volume) because of her worsening psychiatric symptoms, although frequent urinary incontinence and severe urinary retention requiring urethral catheterization suggested the presence of underactive detrusor.

## Conclusions

To our knowledge, this is the first report to describe a 60-year-old female patient with chronic schizophrenia found to have developed possible MSA-P. Although MSA-P comorbidity in patients with schizophrenia may be very rare, MSA-P should be considered when such patients present with worsening and progression of parkinsonism, especially when accompanied by autonomic dysfunction or cerebellar ataxia. In this case, the combination of SPECT imaging and brain MRI, as well as her clinical features, helped us to discriminate MSA-P from the other parkinsonian syndromes in our differential diagnosis.

## Abbreviations

CBD: Corticobasal degeneration
EPS: Extrapyramidal symptoms
LAI: Long-acting injectable
mECT: Modified electroconvulsive therapy
MIBG: 123I-meta-iodobenzylguanidine
MPC: Miyagi Prefecture Center
MRI: Magnetic resonance imaging
MSA: Multiple system atrophy
MSA-C: Multiple system atrophy with predominant cerebellar features
MSA-P: Multiple system atrophy with predominant parkinsonism
PSP: Progressive supranuclear palsy
SPECT: Single-photon emission computed tomography
T2WI: T2-weighted magnetic resonance images

## References

[1] Gilman S, Wenning GK, Low PA, Brooks DJ, Mathias CJ, Trojanowski JQ (2008). Second consensus statement on the diagnosis of multiple system atrophy. Neurology.

[2] Bower JH, Maraganore DM, McDonnell SK, Rocca WA (1997). Incidence of progressive supranuclear palsy and multiple system atrophy in Olmsted County, Minnesota, 1976 to 1990. Neurology.

[3] Schrag A, Ben-Shlomo Y, Quinn NP (1999). Prevalence of progressive supranuclear palsy and multiple system atrophy: a cross-sectional study. Lancet.

[4] Wenning GK, Colosimo C, Geser F, Poewe W (2004). Multiple system atrophy. Lancet Neurol.

[5] Vanacore N (2005). Epidemiological evidence on multiple system atrophy. J Neural Transm (Vienna).

[6] Bjornsdottir A, Gudmundsson G, Blondal H, Olafsson E (2013). Incidence and prevalence of multiple system atrophy: a nationwide study in Iceland. J Neurol Neurosurg Psychiatry.

[7] Ciolli L, Krismer F, Nicoletti F, Wenning GK. An update on the cerebellar subtype of multiple system atrophy. Cerebellum Ataxias. 2014;1:14. 10.1186/s40673-014-0014-7 PMC4552412 26331038

[8] Fanciulli A, Wenning GK (2015). Multiple-system atrophy. N Engl J Med.

[9] Watanabe H, Saito Y, Terao S, Ando T, Kachi T, Mukai E (2002). Progression and prognosis in multiple system atrophy: an analysis of 230 Japanese patients. Brain.

[10] Yabe I, Soma H, Takei A, Fujiki N, Yanagihara T, Sasaki H (2006). MSA-C is the predominant clinical phenotype of MSA in Japan: analysis of 142 patients with probable MSA. J Neurol Sci.

[11] Owen MJ, Sawa A, Mortensen PB (2016). Schizophrenia. Lancet.

[12] Mueser KT, McGurk SR (2004). Schizophrenia. Lancet.

[13] Leucht S, Cipriani A, Spineli L, Mavridis D, Orey D, Richter F (2013). Comparative efficacy and tolerability of 15 antipsychotic drugs in schizophrenia: a multiple-treatments meta-analysis. Lancet.

[14] Dejerine J, Thomas A (1900). L’ atrophie olivo-ponto-cérébelleuse. Nouvelle iconographie de la Salpêtrière.

[15] Shy GM, Drager GA (1960). A neurological syndrome associated with orthostatic hypotension: a clinical-pathologic study. Arch Neurol.

[16] Van Eecken HAR (1960). Van Bogaert, L Striatopallidal-nigral degeneration. J Neuropath Exp Neurol.

[17] Papp MI, Kahn JE, Lantos PL (1989). Glial cytoplasmic inclusions in the CNS of patients with multiple system atrophy (striatonigral degeneration, olivopontocerebellar atrophy and shy-Drager syndrome). J Neurol Sci.

[18] Wakabayashi K, Yoshimoto M, Tsuji S, Takahashi H (1998). Alpha-synuclein immunoreactivity in glial cytoplasmic inclusions in multiple system atrophy. Neurosci Lett.

[19] Goedert M, Jakes R, Spillantini MG (2017). The Synucleinopathies: twenty years on. J Parkinsons Dis.

[20] Gilman S, Low PA, Quinn N, Albanese A, Ben-Shlomo Y, Fowler CJ (1998). Consensus statement on the diagnosis of multiple system atrophy. J Auton Nerv Syst.

[21] Low PA, Reich SG, Jankovic J, Shults CW, Stern MB, Novak P (2015). Natural history of multiple system atrophy in the USA: a prospective cohort study. Lancet Neurol.

[22] Kim HJ, Jeon BS, Lee JY, Yun JY (2011). Survival of Korean patients with multiple system atrophy. Mov Disord.

[23] Ben-Shlomo Y, Wenning GK, Tison F, Quinn NP (1997). Survival of patients with pathologically proven multiple system atrophy: a meta-analysis. Neurology.

[24] Wenning GK, Geser F, Krismer F, Seppi K, Duerr S, Boesch S (2013). The natural history of multiple system atrophy: a prospective European cohort study. Lancet Neurol.

[25] Schrag A, Wenning GK, Quinn N, Ben-Shlomo Y (2008). Survival in multiple system atrophy. Mov Disord.

[26] Shimohata TOT, Nakayama H, Tomita M, Shinoda H, Nishizawa M (2008). Frequency of nocturnal sudden death in patients with multiple system atrophy. J Neurol.

[27] Fereshtehnejad SM, Lökk J (2014). Orthostatic hypotension in patients with Parkinson's disease and atypical parkinsonism. Parkinsons Dis.

[28] Pavy-Le Traon A, Piedvache A, Perez-Lloret S, Calandra-Buonaura G, Cochen-De Cock V, Colosimo C (2016). New insights into orthostatic hypotension in multiple system atrophy: a European multicentre cohort study. J Neurol Neurosurg Psychiatry.

[29] Sun Z, Jia D, Shi Y, Hou X, Yang X, Guo J (2016). Prediction of orthostatic hypotension in multiple system atrophy and Parkinson disease. Sci Rep.

[30] Sakakibara R, Hattori T, Uchiyama T, Kita T, Asahina M, Suzuki A (2000). Urinary dysfunction and orthostatic hypotension in multiple system atrophy: which is the more common and earlier manifestation?. J Neurol Neurosurg Psychiatry.

[31] Wenning GK, Shephard B, Hawkes C, Petruckevitch A, Lees A, Quinn N (1995). Olfactory function in atypical parkinsonian syndromes. Acta Neurol Scand.

[32] Haehner A, Hummel T, Reichmann H (2009). Olfactory dysfunction as a diagnostic marker for Parkinson's disease. Expert Rev Neurother.

[33] Kikuchi A, Baba T, Hasegawa T, Sugeno N, Konno M, Takeda A (2011). Differentiating Parkinson's disease from multiple system atrophy by [123I] meta-iodobenzylguanidine myocardial scintigraphy and olfactory test. Parkinsonism Relat Disord.

[34] Garland EM, Raj SR, Peltier AC, Robertson D, Biaggioni I (2011). A cross-sectional study contrasting olfactory function in autonomic disorders. Neurology.

[35] Suzuki M, Hashimoto M, Yoshioka M, Murakami M, Kawasaki K, Urashima M (2011). The odor stick identification test for Japanese differentiates Parkinson's disease from multiple system atrophy and progressive supra nuclear palsy. BMC Neurol.

[36] Doty RL (2012). Olfactory dysfunction in Parkinson disease. Nat Rev Neurol.

[37] Krismer F, Pinter B, Mueller C, Mahlknecht P, Nocker M, Reiter E (2017). Sniffing the diagnosis: olfactory testing in neurodegenerative parkinsonism. Parkinsonism Relat Disord.

[38] Goldstein DS, Holmes C, Bentho O, Sato T, Moak J, Sharabi Y (2008). Biomarkers to detect central dopamine deficiency and distinguish Parkinson disease from multiple system atrophy. Parkinsonism Relat Disord.

[39] Fujita H, Suzuki K, Numao A, Watanabe Y, Uchiyama T, Miyamoto T (2016). Usefulness of cardiac MIBG scintigraphy, olfactory testing and substantia Nigra Hyperechogenicity as additional diagnostic markers for distinguishing between Parkinson's disease and atypical parkinsonian syndromes. PLoS One.

[40] Constantinescu R, Richard I, Kurlan R (2007). Levodopa responsiveness in disorders with parkinsonism: a review of the literature. Mov Disord.

[41] Wenning GK, Tison F, Ben Shlomo Y, Daniel SE, Quinn NP (1997). Multiple system atrophy: a review of 203 pathologically proven cases. Mov Disord.

[42] Feng JY, Huang B, Yang WQ, Zhang YH, Wang LM, Wang LJ (2015). The putaminal abnormalities on 3.0T magnetic resonance imaging: can they separate parkinsonism-predominant multiple system atrophy from Parkinson's disease?. Acta Radiol.

[43] Massey LA, Micallef C, Paviour DC, O'Sullivan SS, Ling H, Williams DR (2012). Conventional magnetic resonance imaging in confirmed progressive supranuclear palsy and multiple system atrophy. Mov Disord.

[44] Taki J, Yoshita M, Yamada M, Tonami N (2004). Significance of 123I-MIBG scintigraphy as a pathophysiological indicator in the assessment of Parkinson's disease and related disorders: it can be a specific marker for Lewy body disease. Ann Nucl Med.

[45] Raffel DM, Koeppe RA, Little R, Wang CN, Liu S, Junck L (2006). PET measurement of cardiac and nigrostriatal denervation in parkinsonian syndromes. J Nucl Med.

[46] Jin S, Oh M, Oh SJ, Lee SJ, Chung SJ, Lee CS (2013). Differential diagnosis of parkinsonism using dual-phase F-18 FP-CIT PET imaging. Nucl Med Mol Imaging.

[47] Mateos JJ, Lomeña F, Parellada E, Font M, Fernandez E, Pavia J (2005). Decreased striatal dopamine transporter binding assessed with [123I] FP-CIT in first-episode schizophrenic patients with and without short-term antipsychotic-induced parkinsonism. Psychopharmacology.

[48] Mateos JJ, Lomeña F, Parellada E, Font M, Fernández E, Pavia J (2006). Striatal dopamine transporter density decrease in first episode schizophrenic patients treated with risperidone. Rev Esp Med Nucl.

[49] Mateos JJ, Lomeña F, Parellada E, Mireia F, Fernandez-Egea E, Pavia J (2007). Lower striatal dopamine transporter binding in neuroleptic-naive schizophrenic patients is not related to antipsychotic treatment but it suggests an illness trait. Psychopharmacology.

[50] Tinazzi M, Ottaviani S, Isaias IU, Pasquin I, Steinmayr M, Vampini C (2008). [123I]FP-CIT SPET imaging in drug-induced parkinsonism. Mov Disord.

[51] Tinazzi M, Antonini A, Bovi T, Pasquin I, Steinmayr M, Moretto G (2009). Clinical and [123I]FP-CIT SPET imaging follow-up in patients with drug-induced parkinsonism. J Neurol.

[52] Tinazzi M, Cipriani A, Matinella A, Cannas A, Solla P, Nicoletti A (2012). [^123^I]FP-CIT single photon emission computed tomography findings in drug-induced parkinsonism. Schizophr Res.

[53] Tinazzi M, Morgante F, Matinella A, Bovi T, Cannas A, Solla P (2014). Imaging of the dopamine transporter predicts pattern of disease progression and response to levodopa in patients with schizophrenia and parkinsonism: a 2-year follow-up multicenter study. Schizophr Res.

[54] Kurata T, Kametaka S, Ohta Y, Morimoto N, Deguchi S, Deguchi K (2011). PSP as distinguished from CBD, MSA-P and PD by clinical and imaging differences at an early stage. Intern Med.

[55] Boxer AL, Geschwind MD, Belfor N, Gorno-Tempini ML, Schauer GF, Miller BL (2006). Patterns of brain atrophy that differentiate corticobasal degeneration syndrome from progressive supranuclear palsy. Arch Neurol.

[56] Okada N, Fukunaga M, Yamashita F, Koshiyama D, Yamamori H, Ohi K (2016). Abnormal asymmetries in subcortical brain volume in schizophrenia. Mol Psychiatry.

[57] Van Erp TG, Hibar DP, Rasmussen JM, Glahn DC, Pearlson GD, Andreassen OA (2016). Subcortical brain volume abnormalities in 2028 individuals with schizophrenia and 2540 healthy controls via the ENIGMA consortium. Mol Psychiatry.

[58] Hooten WM, Melin G, Richardson JW (1998). Response of the parkinsonian symptoms of multiple system atrophy to ECT. Am J Psychiatry.

[59] Roane DM, Rogers JD, Helew L, Zarate J (2000). Electroconvulsive therapy for elderly patients with multiple system atrophy: a case series. Am J Geriatr Psychiatry.

[60] Parsa MA, Simon M, Dubrow C, Ramirez LF, Meltzer HY (1993). Psychiatric manifestations of olivo-ponto-cerebellar atrophy and treatment with clozapine. Int J Psychiatry Med.

[61] Ehrt U, Brieger P, Broich K, Marneros A (1999). Psychotic symptoms as initial manifestation of a multiple system atrophy. Fortschr Neurol Psychiatr.

[62] Benrud-Larson LM, Sandroni P, Schrag A, Low PA (2005). Depressive symptoms and life satisfaction in patients with multiple system atrophy. Mov Disord.

[63] Duggal HS (2005). Cognitive affective psychosis syndrome in a patient with sporadic olivopontocerebellar atrophy. J Neuropsychiatry Clin Neurosci.

[64] Malone D, Dennis MS (2005). Multiple system atrophy and hallucinations--a short report. Int J Geriatr Psychiatry.

[65] Papapetropoulos S, Tuchman A, Laufer D, Mash DC (2007). Hallucinations in multiple system atrophy. Parkinsonism Relat Disord.

[66] Schrag A, Sheikh S, Quinn NP, Lees AJ, Selai C, Mathias C (2010). A comparison of depression, anxiety, and health status in patients with progressive supranuclear palsy and multiple system atrophy. Mov Disord.

[67] Zhang LY, Cao B, Zou YT, Wei QQ, Ou RW, Zhao B, et al. Depression and anxiety in multiple system atrophy. Acta Neurol Scand. 2017;137:33–7. 10.1111/ane.12804 28748633

[68] Tkachev D, Mimmack ML, Ryan MM, Wayland M, Freeman T, Jones PB (2003). Oligodendrocyte dysfunction in schizophrenia and bipolar disorder. Lancet.

[69] Takahashi N, Sakurai T, Davis KL, Buxbaum JD (2011). Linking oligodendrocyte and myelin dysfunction to neurocircuitry abnormalities in schizophrenia. Prog Neurobiol.

[70] Miyata S, Hattori T, Shimizu S, Ito A, Tohyama M (2015). Disturbance of oligodendrocyte function plays a key role in the pathogenesis of schizophrenia and major depressive disorder. Biomed Res Int.

[71] Spokes EG, Bannister R, Oppenheimer DR (1979). Multiple system atrophy with autonomic failure: clinical, histological and neurochemical observations on four cases. J Neurol Sci.

